# Negotiating cultural voice in the platform era: the access–representation nexus in Asia-Pacific screen production

**DOI:** 10.3389/fsoc.2026.1987870

**Published:** 2026-10-14

**Authors:** Apinya Anphanlam

**Affiliations:** Department of Performing Arts, Faculty of Fine-Applied Arts and Cultural Science, Mahasarakham University, Mahasarakham, Thailand

**Keywords:** access-representation nexus, Asia-Pacific, cultural voice, platformization, production governance, SVoD, transnational co-production

## Abstract

**Introduction:**

This study examines how screen producers navigate cultural representation, market access, and creative authority within transnational production governance in the platform era. Moving beyond a binary opposition between platform dominance and producer autonomy, it develops the Access–Representation Nexus as a conceptual framework for examining the relationship between financial, legal, institutional, and distributive access and producers’ ability to retain authority over culturally consequential representation.

**Methods:**

The study employed a qualitative multi-method design combining semi-structured interviews with ten experienced industry informants from Thailand, the Philippines, and Australia with comparative analysis of the cross-border productions *TWIN* and *The Medium*.

**Results:**

The findings identify a recurrent “negotiate–adapt” dynamic involving territorial-rights protection, negotiated creative authority, manufactured cultural proximity, and selective narrative adaptation. Importantly, the data suggest that the principal governance mechanisms identified—rights allocation, territorial licensing, financing conditions, distribution arrangements, and creative-authority settlements—are not specific to digital platforms. These mechanisms predate streaming and operate through co-production, broadcasting, studio, financing, and public-institutional arrangements. Platformization is therefore understood as a broader structural context that may intensify or reconfigure existing production-governance relationships rather than originate them.

**Discussion:**

The access–representation nexus conceptualizes market access and representational authority as analytically distinct but interdependent dimensions of production negotiation, showing how producers may concede economic, territorial, or contractual rights while retaining authority over culturally consequential creative decisions. Cultural agency is therefore understood as relational, negotiated, and structurally conditioned rather than as either complete autonomy or straightforward subordination.

## 1 Introduction

The global audiovisual industry has undergone substantial restructuring as territorial pre-sales, theatrical distribution, and localized release windows have become increasingly intertwined with over-the-top (OTT) video-on-demand services, subscription video-on-demand (SVoD) platforms, and transnational financing and distribution arrangements (Lotz, 2021). Across contemporary screen industries, digital platforms have become important intermediaries in financing, commissioning, distribution, audience access, and cultural circulation, while operating alongside established broadcasters, studios, distributors, public agencies, and co-production systems (Gambarato and Heuman, 2024; Hesmondhalgh et al., 2023; Jin, 2025; Jin et al., 2023). Platformization is therefore treated here as part of a changing institutional environment within which screen producers seek financing, negotiate rights, organize distribution, and position culturally specific content for transnational circulation (Couldry and Mejias, 2020; Evens and Donders, 2018; Li, 2022; van Dijck et al., 2018).

However, the institutional mechanisms through which screen production is governed—including territorial licensing, intellectual-property allocation, co-financing, distribution-rights agreements, exclusivity, and negotiated creative authority—should not be treated as originating with digital platforms. Such mechanisms also operate through broadcasters, studios, financiers, public funding systems, treaty arrangements, and international co-productions. Platformization is therefore understood here not as the source of these governance mechanisms but as a structural context that can intensify and reconfigure their operation, including the scale and timing of rights acquisition, the pathways through which content reaches transnational markets, and the strategic importance of access to circulation (Evens and Donders, 2018; Nieborg and Poell, 2018; Idiz and Poell, 2025; Stelmach, 2026). This distinction is important because platform influence cannot be inferred automatically from the presence of financing agreements, territorial licensing, intellectual-property negotiations, or creative bargaining.

The distinction also reframes the question of producer agency. Greater access to transnational finance, distribution, and circulation does not necessarily correspond to equivalent authority over culturally consequential creative decisions. Producers may concede economic or territorial rights while retaining authority over narrative voice, final decision-making, culturally significant meanings, or other creative domains. Conversely, economic ownership does not necessarily guarantee equivalent representational authority. Existing work on platformization and political economy has demonstrated the structural asymmetries created by concentrated infrastructural and market power (Couldry and Mejias, 2020; Evens and Donders, 2018; Nieborg and Poell, 2018; van Dijck et al., 2018), while field theory directs attention to how actors occupy unequal positions according to their access to different forms of economic, cultural, and symbolic capital (Bourdieu, 1993, 2005). The issue, therefore, is not simply whether producers possess or lack autonomy, but how different forms of authority are negotiated within unequal production relationships.

This paper conceptualizes this relationship as the Access–Representation Nexus. Access architecture refers to the financial, legal, institutional, territorial, and distribution arrangements through which a production obtains resources and pathways to markets. Representation control refers to the differentiated forms of authority through which producers retain, share, or concede control over narrative voice, culturally significant meanings, creative decision-making, adaptation, and related representational domains. The Nexus treats these dimensions as analytically distinct but relational: gaining access to finance or circulation may involve concessions in particular domains of authority, while cultural knowledge, professional legitimacy, regional networks, and symbolic capital may strengthen producers’ positions in negotiation. In this sense, cultural agency is approached as relational and structurally conditioned rather than as either complete autonomy or straightforward subordination (Bhabha, 2012; Bourdieu, 1993, 2005).

The analytical concern of this study is therefore not whether platforms independently cause contractual or creative outcomes. Instead, it examines how producers negotiate access and representational authority within transnational production-governance arrangements that operate in a platformized media environment. Platforms constitute important actors within this environment, but they coexist with producers, broadcasters, financiers, distributors, public agencies, and co-production partners whose governance practices cannot be reduced to platform intervention. This distinction is especially important for the present study because its empirical evidence concerns producers’ and institutional actors’ experiences of financing, rights allocation, co-production, distribution, and creative negotiation rather than direct observation of proprietary platform algorithms, audience analytics, or internal platform decision-making. Accordingly, the study asks: How do screen producers negotiate market access and representational authority within transnational production governance in the platform era?

The paper argues that producers engage in a recurring “negotiate–adapt” dynamic in which rights protection, culturally grounded resources, relationship building, and selective adaptation are mobilized to navigate unequal production environments. The proposed Access–Representation Nexus therefore shifts attention from a binary opposition between platform domination and producer autonomy toward the institutional conditions under which economic, contractual, territorial, and representational forms of authority are negotiated. Rather than treating platformization as the origin of these mechanisms, the framework examines how pre-existing production-governance arrangements are intensified, reconfigured, and strategically navigated under contemporary conditions of transnational circulation.

## 2 Materials and methods

### 2.1 Research design and analytical framework

To investigate how screen producers negotiate market access, creative authority, and cultural voice within transnational production arrangements, this study employed a qualitative multi-method design combining comparative case analysis of two cross-border screen productions, *TWIN* and *The Medium*, with semi-structured interviews with ten industry informants from Thailand, the Philippines, and Australia. The empirical object of the study is conceptualized as production governance in the platform era: the institutional, contractual, financial, territorial, and creative arrangements through which production resources, rights, market access, and decision-making authority are allocated.

Platformization is treated as a broader structural context rather than an independently observed causal mechanism. Existing research demonstrates that digital platforms have acquired significant infrastructural and intermediary power in commissioning, distribution, audience access, data, and cultural circulation (Couldry and Mejias, 2020; van Dijck et al., 2018). However, the present study does not assume that the contractual and institutional mechanisms observed in the empirical material originate with platforms. Financing arrangements, intellectual-property allocation, territorial licensing, exclusivity, distribution rights, and creative-authority settlements also operate within broadcaster, studio, co-production, and other established production systems (Evens and Donders, 2018; Nieborg and Poell, 2018; Idiz and Poell, 2025; Stelmach, 2026). We understand platformization as a condition that may intensify or reconfigure existing governance relationships rather than as their sole source.

This distinction also reflects the evidentiary boundaries of the study. The empirical material provides evidence concerning producers’ and institutional actors’ experiences of financing, rights allocation, transnational co-production, distribution, and creative negotiation, including interactions with global streaming services. It does not provide direct access to proprietary platform algorithms, internal audience analytics, confidential platform contracts, or platform executives. Accordingly, claims concerning platform-specific internal governance are not inferred solely from producers’ retrospective accounts.

The analytical framework combines critical political economy, Bourdieu’s field theory, and Bhabha’s concept of the Third Space. Within this framework, platforms are treated as one category of actor within a wider production field that also includes producers, studios, broadcasters, financiers, distributors, public agencies, and co-production partners. Bourdieu’s field theory is particularly useful for conceptualizing these actors as occupying unequal positions according to their access to economic, cultural, and symbolic capital, which in turn shapes their relative negotiating capacities (Bourdieu, 1993, 2005).

The Access–Representation Nexus organizes the analysis around two interrelated dimensions. Access architecture refers to the financial, legal, institutional, territorial, and distribution conditions through which productions obtain resources and pathways to markets. These include financing arrangements, intellectual-property allocation, exclusivity, territorial licensing, distribution rights, public incentives, institutional relationships, and distribution pathways (Evens and Donders, 2018; Idiz and Poell, 2025; Stelmach, 2026). Representation control refers to the differentiated allocation of authority over culturally consequential creative decisions, including narrative voice, final decision-making, adaptation, language, casting, localization, and the preservation or modification of culturally specific meanings (Nieborg and Poell, 2018; Kim, 2022; Valtysson, 2022).

Rather than constituting a separate third dimension, negotiated agency is treated as the relational process through which producers move between these two domains. Producers may accept modification to, or concession in, financial, territorial, or formal aspects of a project while attempting to retain authority over culturally significant meanings, narrative principles, creative decisions, or strategically important rights. This understanding draws on Bhabha’s concept of the Third Space and Hall’s concept of negotiated meaning, both of which emphasize cultural negotiation as neither complete resistance nor simple submission (Bhabha, 2012; Hall, 2019).

The framework therefore does not assume that territorial licensing, IP negotiation, financing conditions, or creative-authority arrangements originate with digital platforms. Instead, it examines how these longer-standing production-governance mechanisms are negotiated under contemporary conditions of platformization and transnational circulation. The Access–Representation Nexus consequently provides the analytical structure for case comparison, interview design, coding, and interpretation by examining how conditions of access interact with the distribution of representational authority.

### 2.2 Empirical context and comparative case selection

The empirical corpus consisted primarily of semi-structured interview transcripts and structured interview materials generated with industry participants from Thailand, the Philippines, and Australia. Within this broader corpus, two cross-border productions, *TWIN* and *The Medium*, were selected for focused comparative analysis because the interviews provided sufficiently detailed project-specific evidence concerning financing, production collaboration, territorial and intellectual-property arrangements, creative decision-making, cultural adaptation, and distribution strategy.

The two cases were selected analytically rather than as statistically representative examples. Their comparative value lies in the contrasting production-governance configurations they provide. *TWIN* represents a Thailand–Singapore commercial television co-production involving cross-border financing, institutional support, territorial allocation, and explicitly negotiated creative authority. *The Medium* represents a Thailand–South Korea feature-film co-production in which regional collaboration, Thai-led script development, production responsibility, intellectual-property interests, and territorial distribution formed a different configuration of access and creative authority.

Four dimensions guide the case comparison: (1) access to financing and institutional support; (2) intellectual-property and territorial-rights arrangements; (3) the allocation of creative decision-making authority; and (4) the preservation, negotiation, or adaptation of culturally consequential narrative elements. These dimensions correspond to the broader Access–Representation Nexus and enable the cases to be compared as contrasting configurations of access architecture and representation control rather than as representative samples of the Asia-Pacific screen sector.

*TWIN* was selected because the interview with a principal creative decision-maker provided detailed evidence extending from project negotiation and institutional support to screenplay development, casting, production oversight, creative authority, distribution, and territorial-rights allocation. The case therefore enables close examination of how transnational financing and market expansion coexist with negotiated local creative authority.

*The Medium* was selected because an interview with a senior industry representative directly involved in international sales, distribution, and collaboration provided project-specific evidence regarding the production partnership, script development, intellectual-property priorities, territorial distribution, and broader co-production strategy. The case provides a contrasting intra-Asian production configuration in which international collaboration operated alongside substantial Thai participation in script development and production governance.

Evidence from the two cases was interpreted alongside the wider interview corpus. The cases therefore serve as analytically illustrative examples for examining contrasting configurations of access and representational authority, rather than as statistically representative cases from which claims about the Asia-Pacific region can be inferred.

### 2.3 Informant selection and sample adequacy

Ten industry informants from Thailand, the Philippines, and Australia were recruited through criterion-based purposive sampling with limited referral-based extension. The aim was not statistical representation but the selection of information-rich participants who had direct experience relevant to the study’s focus on market access, production governance, rights allocation, creative authority, and transnational screen collaboration. Participants were required to meet three core criteria: (1) substantial professional experience in the screen sector, generally a minimum of eight to ten years; (2) direct executive, creative, technical, legal, production, or policy involvement in international screen production; and (3) direct experience with at least one of the following: cross-border co-production, transnational financing, international distribution, or engagement with global SVoD services.

Recruitment sought analytical variation across positions in the production field rather than numerical balance between national groups. The final sample included independent producers and directors, commercial studio executives, cross-border production and legal specialists, technical practitioners, and governmental or media-policy actors. This variation enabled comparison across creative, commercial, contractual, technical, and institutional perspectives. Participants were assigned anonymous identifiers from KI-01 to KI-10, and their professional roles, industry experience, field positions, and principal analytical relevance are summarized in Table 1.

**Table 1:** Summary matrix of industry key informants across four sample tiers (*n* = 10).

Code	Country	Field position	Professional role	Experience	Analytical role
KI-01	Thailand	Tier 1 (Indie)	Independent Film Producer	15 + years	European fund pitching & IP preservation
KI-02	Philippines	Tier 1 (Indie)	Feature Director & Producer	12 years	SVoD acquisitions & local narrative legibility
KI-03	Thailand	Tier 2 (Studio)	Network Showrunner / Executive	20 + years	Commercial co-productions (*TWIN*)
KI-04	Thailand	Tier 2 (Studio)	Studio Executive Producer	14 years	Intra-Asian co-financing & distribution splits
KI-05	Australia	Tier 3 (Line)	Co-Production Legal Specialist	18 years	Trans-Pacific legal & tax incentive architecture
KI-06	Philippines	Tier 1 (Indie)	Independent Line Producer	10 years	Festival-to-platform pipelines & precarity
KI-07	Thailand	Tier 3 (Line)	Post-Production Supervisor	11 years	Platform localization & technical standardization
KI-08	Australia	Tier 4 (State)	Former Screen Commission Officer	16 years	Bilateral treaties & soft-power policy
KI-09	Philippines	Tier 4 (State)	Media Policy Consultant	13 years	Platform regulation & creator labor rights
KI-10	Thailand	Tier 1 (Indie)	Genre Director / Creator	15 years	Regional genre mechanics (*The Medium*) & SVoD deals

The adequacy of the sample was assessed according to information power rather than sample size alone (Malterud et al., 2016). A smaller qualitative sample can provide sufficient analytical material when the research question is focused, participants possess highly specific knowledge, the interviews are information dense, and the analysis is conducted in depth across theoretically relevant positions. In this study, the informants were senior or experienced practitioners whose professional roles provided direct access to negotiation processes concerning financing, territorial rights, intellectual property, distribution, creative authority, cultural adaptation, and institutional support. The combination of highly specific expertise and cross-role comparison therefore increased the information power of the sample.

Recruitment and analysis were also treated as iterative rather than entirely sequential. Early interviews with producers and industry practitioners foregrounded not only creative and contractual negotiation but also the importance of institutional incentives, legal structures, and policy environments. Subsequent recruitment was therefore broadened to include legal, production-support, governmental, and media-policy perspectives so that emerging institutional themes could be examined from additional field positions. Referral-based recruitment was used only where potential participants continued to meet the predetermined inclusion criteria; referrals did not replace purposive selection.

Recruitment ceased when additional interviews were no longer substantially extending the principal analytical categories relevant to the study, particularly those concerning rights and territorial arrangements, financing and institutional access, creative decision-making, cultural negotiation, and distribution pathways. This criterion was treated as analytical sufficiency rather than numerical saturation. Divergent and contradictory accounts were retained during coding and comparison rather than excluded, because variation in negotiating position was itself analytically important to the Access–Representation Nexus.

Analytical sufficiency can be demonstrated with reference to the coding record. All thirty-four first-order codes in the final coding frame had been established by the eighth interview; the final two interviews contributed additional instances, refinements of code boundaries, and institutional detail, but no new first-order codes and no new second-order themes (see Supplementary Table S3). The final interviews were therefore treated as consolidating rather than expanding the analytical structure, which we report here as the empirical basis for the sufficiency judgement rather than as a claim of exhaustive thematic saturation.

### 2.4 Data elicitation and collection

Data was collected over the course of eighteen months. Semi-structured interviews were conducted either in-person at local film-market locations or over secure video conferencing, lasting approximately 60 to 90 min. The interviews were recorded, transcribed, anonymized, and verified for accuracy with interviewees’ permission.

Four modules from the analytical framework served as the theoretical foundation for the interview protocol as shown in Table 2.

**Table 2:** Analytical framework for the semi-structured interview protocol.

Protocol module	Analytical focus	Principal themes
1. Platform and field re-intermediation	Structural transformation of screen production	Platformization, financing dependence, distribution, precarity, changing intermediary relations
2. Access architecture	Financial and legal conditions of participation	IP acquisition, licensing, exclusivity, holdbacks, territorial rights, revenue arrangements
3. Representation control	Conditions governing cultural and creative authority	Cultural legibility, final cut, localization, narrative modification, authentic versus curated localism
4. Negotiation strategies/negotiated agency	Producer responses to platform constraints	Tactical concessions, capital mobilization, cultural translation, regional leverage, protection of cultural meanings

Interviews did not directly solicit confidential contractual documents or commercially sensitive information, as several interviewees were bound by commercial non-disclosure agreements. Consequently, critical-incident and scenario-based questioning was employed to elicit interviewees’ accounts of the financial, contractual, and creative trade-offs that occurred during project pitching, financing, contract negotiation, production, localization, and final editing. This method enabled participants to engage in discussions regarding negotiation processes and decision-making boundaries without disclosing protected contractual information.

### 2.5 Data analysis

The analysis followed a critical-realist, abductive thematic approach. Critical realism provided the epistemological orientation by treating participants’ accounts as situated evidence of underlying institutional, contractual, and cultural mechanisms rather than as direct or exhaustive representations of those mechanisms. Abductive reasoning guided an iterative movement between empirical observations and theoretically informed interpretation. Emerging patterns in the interview material were repeatedly compared with concepts concerning field position, forms of capital, cultural negotiation, and the developing Access–Representation Nexus. Thematic analysis therefore served as the principal analytical strategy, while data-near coding, higher-order thematic grouping, theoretical interpretation, comparative analysis, and peer debriefing formed interconnected stages of the same analytical process (Braun and Clarke, 2022).

The first stage emphasized data-near coding. Interview transcripts and structured interview materials were examined using in-vivo and open coding to retain participants’ professional terminology, reported practices, and negotiation experiences before these materials were organized within higher-order analytical categories. Examples of first-order expressions included “establishing territory,” “acquiring the backend,” and “final say,” together with accounts of retaining domestic rights, protecting narrative principles, or selecting financing sources according to the conditions attached. Codes were compared across interviews and refined where conceptually related practices recurred across professional roles and national contexts.

In the second stage, the first-order codes were grouped through abductive and comparative thematic analysis according to relationships among rights, financing, institutional access, distribution, creative authority, narrative control, and adaptation. This process generated 34 first-order codes organized into nine second-order themes. Five themes constituted Access Architecture: (1) rights and territory settlement; (2) funding and incentive navigation; (3) relational infrastructure; (4) institutional frameworks and gaps; and (5) distribution pathway design. Four themes constituted Representation Control: (1) creative authority instruments; (2) narrative authority and cultural voice; (3) negotiated adaptation; and (4) boundary conditions and negative cases. The resulting analytical hierarchy is presented visually in Figure 1, which makes explicit the progression from empirical interview material to first-order codes, second-order themes, and finally the two aggregate dimensions of the Access–Representation Nexus. Because Figure 1 presents representative first-order codes for readability, the complete mapping of all 34 first-order codes to the nine second-order themes and aggregate dimensions is provided in Supplementary Table S2.

**Figure 1:**
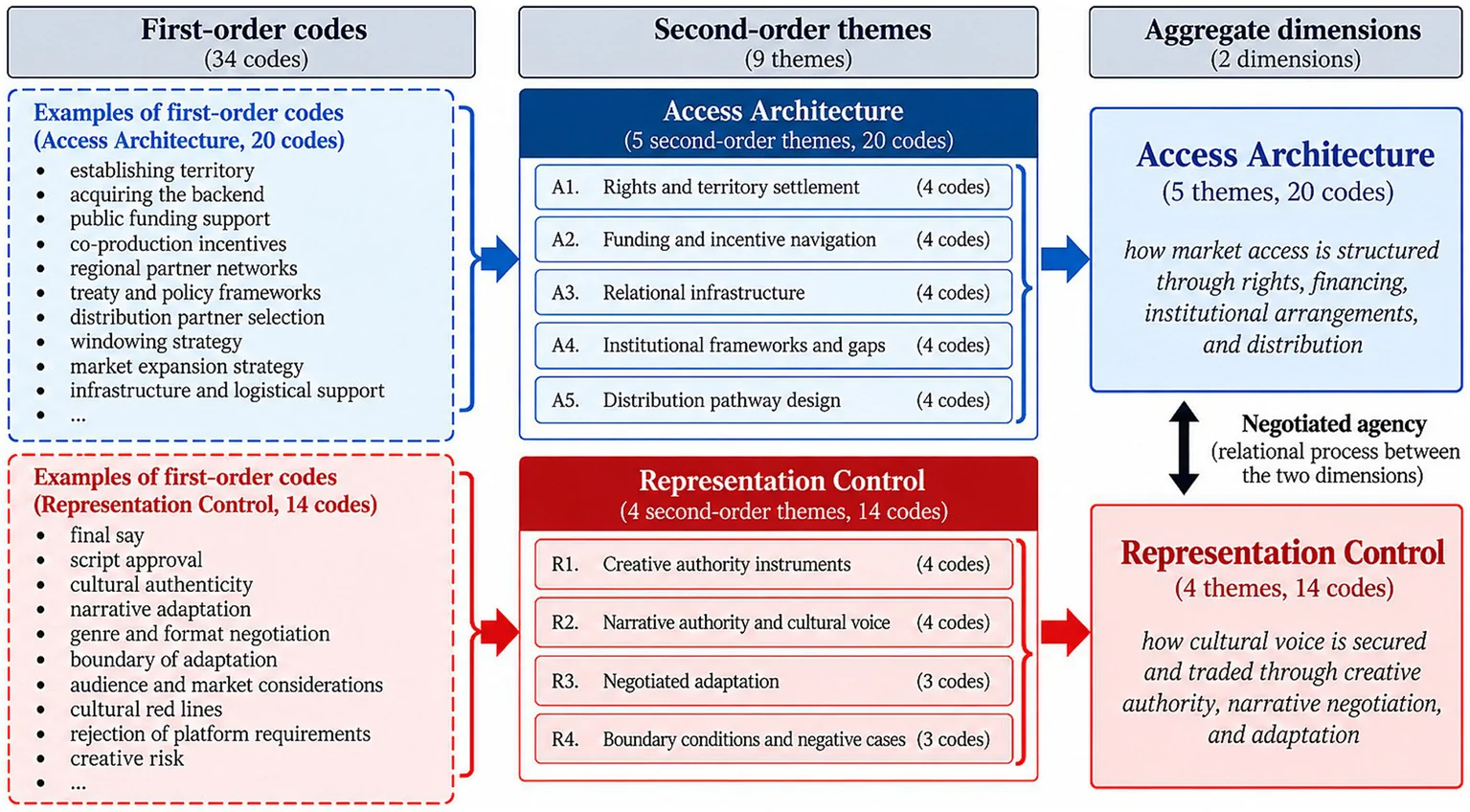
Analytical coding hierarchy of the Access-Representation Nexus. The analytical process moved from 34 first-order codes derived from interview material to nine second-order themes and subsequently to two aggregate dimensions: access architecture (five themes, 20 codes) and representation control (four themes, 14 codes). Negotiated agency is conceptualized as the relational process through which participants navigate the two dimensions. Representative first-order codes are displayed for readability; the complete coding hierarchy is provided in Supplementary Table S2. The figure makes explicit the analytical chain linking empirical interview material to the Access-Representation Nexus.

The third stage involved theoretically informed interpretation. Bourdieu’s concepts of economic, cultural, and symbolic capital were used as sensitizing concepts for interpreting variations in bargaining positions and resource mobilization (Bourdieu, 1993, 2005). Bhabha’s concept of the Third Space and Hall’s concept of negotiated meaning informed the interpretation of selective adaptation and the boundaries between commercial accommodation and the preservation of culturally consequential meanings (Bhabha, 2012; Hall, 2019). These theoretical concepts were not imposed as predetermined first-order coding categories; instead, they were used abductively to interpret relationships emerging from the empirical material.

Negotiated agency was not treated as a separate third aggregate dimension. Rather, it was interpreted as the relational process through which participants navigated Access Architecture and Representation Control. This distinction allowed the analysis to examine how producers could concede financial, territorial, or ownership rights while retaining authority over final decision-making, narrative voice, cultural meaning, or other creative domains.

The coded material was subsequently compared across informant roles, national contexts, and the two production cases, *TWIN* and *The Medium*. Cross-case comparison examined how different configurations of financing, territorial rights, institutional support, distribution, and creative authority produced different negotiated positions within the Access–Representation Nexus. Divergent evidence was retained rather than treated as analytical noise. Negative and boundary cases—including instances in which relational access deteriorated, financing opportunities were rejected, or adherence to cultural principles imposed economic costs—were used to identify conditions under which dominant patterns did not hold.

NVivo was used to organize the hierarchical coding structure, retrieve coded excerpts, compare patterns across informants and cases, and maintain an auditable link between first-order codes, second-order themes, and aggregate dimensions. Interpretive differences were examined through repeated comparison with the source material and peer debriefing. The resulting coding hierarchy is summarized in Figure 1, providing a transparent analytical chain from empirical material to the two aggregate dimensions of the Access–Representation Nexus.

### 2.6 Research rigor and trustworthiness

Evidence triangulation was conducted at the level of the manuscript’s central claims. Claims were compared across informants occupying different creative, commercial, legal, technical, and institutional positions and, where possible, examined against comparative case material and independent documentary or contextual sources. Three evidentiary registers were distinguished: data-supported claims, based on convergent interview and/or documentary evidence; participant-perceived claims, representing reported experiences or interpretations that could not be independently verified; and author-inferred claims, representing theoretical interpretations of empirically supported patterns. Claims concerning confidential contracts, specific rights allocations, or internal platform processes were not treated as independently verified where documentary access was unavailable. Supplementary Table S1 provides the claim-level evidence-triangulation matrix.

Research rigor was addressed through the qualitative criteria of credibility, transferability, dependability, and confirmability (Lincoln and Guba, 1985). Credibility was strengthened through the use of information-rich industry participants, transcript verification, cross-role comparison, cross-case analysis, and peer debriefing. Comparisons among creative, commercial, legal, technical, and institutional actors enabled emerging interpretations to be examined across different positions within the production field rather than being based on a single category of participant.

Dependability and confirmability were supported through an explicit analytical audit trail linking interview material to first-order codes, second-order themes, and the two aggregate dimensions of the Access–Representation Nexus. Coded excerpts were repeatedly checked against their source contexts, and divergent or contradictory material was retained for negative-case analysis rather than removed to achieve thematic consistency. The comparative analysis of *TWIN* and *The Medium* was interpreted alongside the wider interview corpus to distinguish case-specific configurations from patterns recurring across informants.

Transferability was addressed through detailed description of participant roles, case-selection logic, production contexts, and the institutional conditions surrounding the findings. The study does not claim statistical or regional representativeness; instead, it provides sufficient contextual and analytical detail for readers to assess whether the identified relationships may be relevant to other production-governance settings.

### 2.7 Ethical considerations

This research obtained approval from the pertinent Institutional Review Board of Mahasarakham University (approval no. 407-391/2025). All individuals granted informed consent prior to their involvement. Participants were designated only by anonymized identifiers ranging from KI-01 to KI-10, and any personally identifiable or commercially sensitive data was eliminated or obscured in the research findings. Interview recordings, transcripts, and related research documents were securely archived and utilized exclusively for purposes sanctioned by the ethics review procedure.

## 3 Results: the mechanics of the “negotiate–adapt” dynamic

The analysis identified a recurrent “negotiate–adapt” pattern across multiple informants and the two comparative cases. Three closely related practices were especially prominent: the negotiation of rights and territorial arrangements, the mobilization of culturally grounded knowledge and regional relationships, and the selective adaptation of narrative or production elements. These patterns were not inferred from a single participant. Rather, they were identified through comparison across creative, commercial, legal, technical, and institutional positions and were examined against comparative case evidence and documentary or contextual sources where such evidence was available. The evidentiary basis and epistemic status of the principal claims are summarized in Supplementary Table S1.

The three practices were analytically interrelated rather than sequential. Producers negotiated access to financing, institutional support, distribution, and transnational circulation while simultaneously attempting to retain strategically important forms of creative and representational authority. Together, these practices constitute the “negotiate–adapt” dynamic through which participants navigated the relationship between access architecture and representation control examined in Figure 2. The findings therefore describe patterned configurations within the production settings examined rather than universal characteristics of Asia-Pacific screen production.

**Figure 2:**
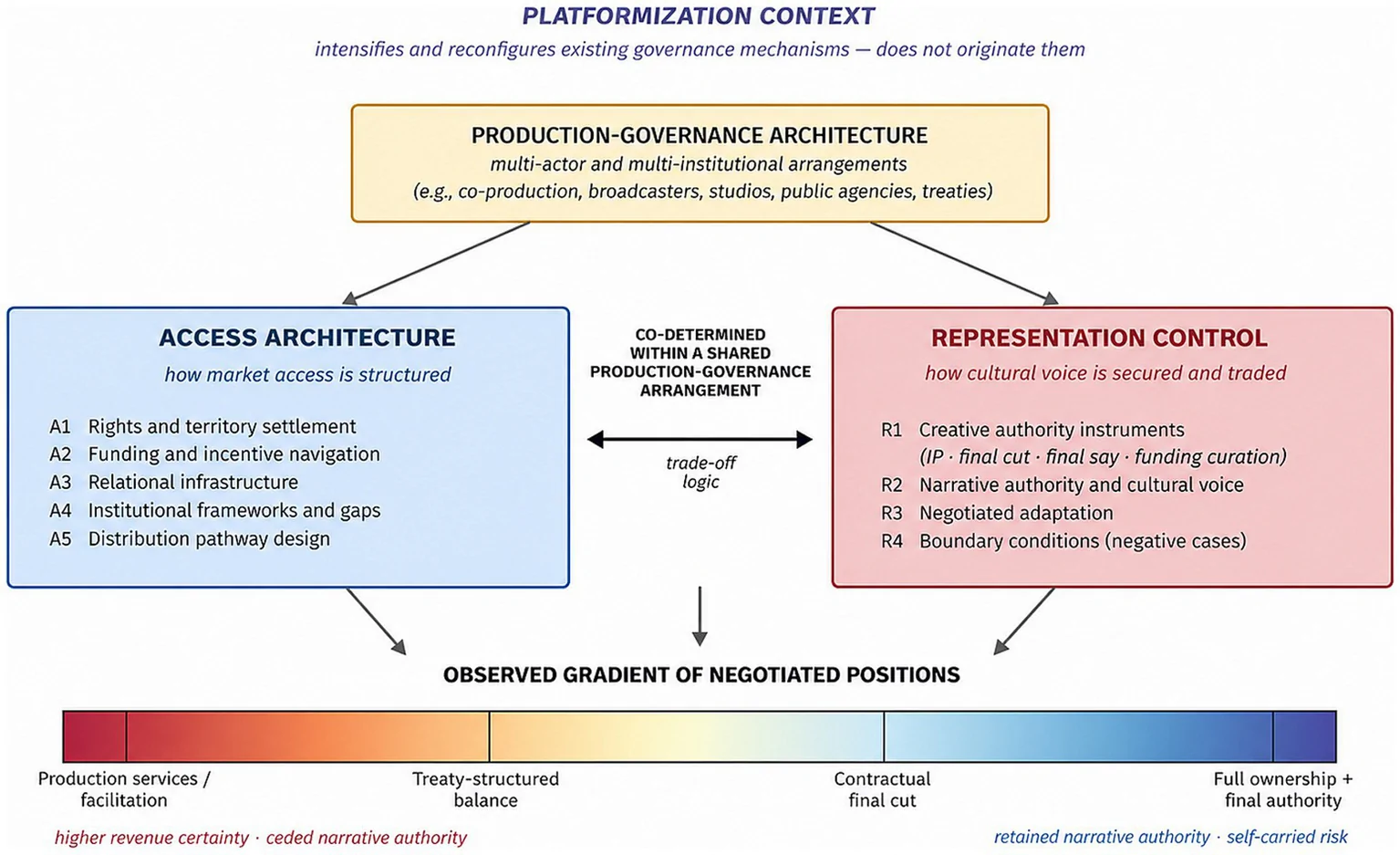
The Access-Representation Nexus. Market access and representational authority are negotiated within a shared production-governance architecture. The wider context of platformization may intensify or reconfigure existing governance mechanisms rather than originate them. Variations in rights, financing, distribution, and creative authority produce an observed gradient of negotiated positions.

In this section, the phrasing of assertions corresponds to their evidential standing. Participant experiences and interpretations are conveyed as participant narratives; project-specific evidence is recognized as case exemplification; and overarching conceptual implications are articulated as interpretive deductions from converging evidence. The relevant categorization of claims as data-supported (DS), participant-perceived/reported (PP), or author-inferred (AI) is detailed in Supplementary Table S1.

### 3.1 Rights architecture as a site of negotiated control

Rights ownership and territorial allocation emerged as recurrent concerns across multiple interviews involving producers, studio practitioners, co-production specialists, and institutional actors. Across these different positions, participants described intellectual property, territorial rights, distribution authority, financing conditions, and creative decision-making as matters that were often negotiated before or alongside financial commitment. The recurrence of this pattern across structurally different informants provides stronger support than any single project account, while official co-production and institutional materials offer contextual corroboration for the broader relationship between financing, territorial participation, and rights allocation (see Supplementary Table S1).

The interviews nevertheless revealed variation in how these arrangements were configured. Independent productions sometimes linked intellectual-property participation to investment shares, whereas commercial producers emphasized territory-specific arrangements, retention of domestic rights, differentiated revenue structures, or early specification of distribution obligations. Participants from the Philippines similarly emphasized the importance of clarifying IP sharing, revenue allocation, and distribution responsibilities at an early stage of collaboration. These differences indicate that access to external financing was usually connected to questions concerning who would control, distribute, and continue to exploit the resulting work. Rather than indicating a single contractual model, the findings point to multiple rights architectures through which market access and continuing authority may be negotiated.

This pattern was also evident at the institutional level. Australian participants described official co-production schemes, producer offsets, regional incentives, and other forms of public support as mechanisms that can strengthen participation in international production by reducing financial barriers and enhancing the attractiveness of jurisdictions or partners. Such institutional arrangements affect producers’ negotiating positions by shaping the resources, incentives, and forms of recognition available within a project. Access architecture should therefore be understood as emerging from the interaction of financing arrangements, co-production frameworks, territorial-rights structures, public institutions, intermediaries, and, where relevant, platform participation rather than as being determined by platforms alone.

#### 3.1.1 IP ownership and territorial ring-fencing

A consistent approach across the interviews was the safeguarding of economically or culturally vital rights, while other rights were shared and distributed by territory. A senior GDH official stated that maintaining intellectual-property ownership and Thai distribution rights is a fundamental negotiating priority for GDH-led co-productions: “If it originates from GDH, we cannot relinquish ownership rights of the intellectual property and the distribution rights in Thailand”.

The same participant characterized territorial distribution as a crucial element of co-production discussions, with national collaborators aiming to maintain control in areas where they had acquired distribution power. This narrative exemplifies a singular method of territorial ring-fencing instead of a universal co-production principle.

The *TWIN* instance offers a pertinent yet different illustration. The interviewed showrunner indicated that rights were distributed significantly by region: the Thai collaborator-maintained control over the Thai market, Singaporean associates managed the Singaporean and Chinese-speaking markets, while profits from other regions were divided post-expense. The situation exemplifies how territorial segmentation can merge international access with continuing control over strategically significant markets.

Numerous autonomous professionals articulated an alternative arrangement wherein intellectual property ownership may be associated with investment involvement, although ultimate creative or editing control resided with the creative team. The evidence across these stories indicates that economic ownership and creative authority can be treated as separate realms rather than inherently linked.

#### 3.1.2 Strategic pre-contractual bargaining

In *TWIN*, the interviewed showrunner reported that final creative authority was agreed upon at an early stage, including authority over major screenplay, casting, and production decisions. This case illustrates a specific mechanism for contractually or institutionally protecting creative authority. Importantly, the broader conclusion that creative authority is multidimensional does not rest on this case alone: other practitioner accounts described different instruments, including IP ownership, final-cut authority, narrative authority, and selective choice of financing sources. The convergent pattern is therefore the differentiation of creative authority across domains rather than the universal use of any single mechanism (see Supplementary Table S1).

### 3.2 Manufactured cultural proximity as tactical capital

Cultural and relational proximity surfaced in several interviews as an asset utilized in project advancement, collaboration establishment, and market strategy. Producers, directors, and institutional stakeholders articulated various yet interconnected types of regional knowledge, encompassing awareness of genre inclinations, enduring professional connections, culturally rooted narrative understanding, and institutional or interpersonal networks. The alignment of these narratives indicates that proximity functions not merely as a trait of the audience but may also serve as a resource in the creation process. The phrase “manufactured cultural proximity” refers to the intentional development and activation of existing cultural and relational connections. It does not suggest that cultural resemblance is inherently fabricated. Instead, producers recognize, cultivate, and convey pertinent cultural associations to enhance cooperation and dissemination. This analysis is corroborated by testimonies from various professional roles and by contextual data about enduring regional collaborations and transnational market demand (refer to Supplementary Table S1). Personal project accounts demonstrate the functioning of this overarching trend. One producer from the Philippines recounted investigating cultural similarities during the scriptwriting process, while other contributors highlighted regional genre connections, ongoing relationship cultivation, and institutionally backed transnational collaborations. These instances represent a cross-interview pattern rather than standalone evidence of market impacts.

#### 3.2.1 Regional identity and intra-Asian resonance

In multiple interviews, respondents indicated that regional cultural awareness is pertinent to project creation, partner selection, and market positioning. A GDH participant noted variations in regional genre inclinations, characterizing Indonesia as especially open to horror narratives rooted in cultural or spiritual themes, while identifying Vietnam as a significant market for comedy and romantic comedy. These findings signify a market evaluation by an industry member rather than empirical evidence of audience reception.

Alternative interview narratives likewise demonstrated the importance of local partners, regional connections, and culturally pertinent knowledge in transnational production. The amalgamation of these narratives indicates that cultural familiarity may function as a productive asset throughout development, collaboration, and dissemination, while the current research does not independently ascertain its impact on audience behavior or market performance.

#### 3.2.2 Hyperlocalism as cultural and market value

Numerous participants characterized culturally distinctive or “hyperlocal” storytelling as a possible means of distinction instead of an intrinsic obstacle to international dissemination. During the TWIN interview, the showrunner expressed a “hyperlocal to global” rationale and pointed out the importance of narratives crafted by producers who are well-acquainted with the regions depicted. He also recognized that international platforms showed a fascination with locally unique material due to its divergence from more conventional American or Korean productions. This assertion is regarded here as a participant’s perception of market demand instead of actual proof of platform strategy.

In several interviews, participants similarly articulated that cultural specificity may coexist with modifications aimed at promoting broader dissemination. TWIN, for instance, integrated more universally comprehensible themes with Thai national identity, while a producer from the Philippines stated that they declined to alter the fundamental cultural essence of a project only to satisfy the demands of a potential financial body.

Collectively, these narratives imply that hyperlocal uniqueness may serve as both cultural differentiation and an asset in specific production discussions. Nonetheless, the research does not independently establish that cultural distinctiveness inherently leads to enhanced bargaining power or market efficacy.

### 3.3 Selective narrative adaptation under transnational legibility

Through several interviews, participants differentiated between aspects they deemed amendable and those they viewed as culturally or creatively immutable. Participants indicated that instead of viewing adaptation and resistance as opposing stances, they deliberately altered certain elements of a production while striving to maintain control over others.

Reported areas of flexibility included casting, traits of the protagonist, stylistic approach to production, market placement, and the expression of ideas designed for cross-market resonance. Conversely, participants often prioritized narrative voice, culturally significant meanings, ultimate decision-making power, and the foundational worldview of a project. The precise distinction between flexible and protected components varied among participants and production contexts, suggesting that adaptation must be understood in terms of what is modified, who authorizes the modification, and which forms of representational authority are maintained.

A Philippine producer, for instance, indicated a preference for seeking alternate finance instead of significantly modifying the cultural essence of a local narrative to meet a potential financial opportunity. This explanation serves as a boundary case demonstrating the possible economic implications of upholding representational ideals, rather than as proof that all producers hold identical views. Supplementary Table S1 encompasses negative and borderline cases, in addition to the convergent data that bolsters the overarching analytical pattern.

#### 3.3.1 The boundary between adaptability and creative authority

The *TWIN* instance exemplifies the coexistence of adaptation and preserved local creative autonomy. The showrunner contact indicated that the main character underwent alterations during the creation phase, the casting process included global cooperation, and the production included themes designed to resonate across several national markets. Simultaneously, the showrunner indicated maintaining ultimate control over significant writing, casting, and production choices. The situation exemplifies a scenario where formal adaptation transpired within clearly defined negotiated creative limits.

Numerous autonomous professionals articulated a pertinent differentiation. They indicated that global funding may affect the production framework and that involvement in intellectual property might be associated with investment, although the final edit, directorial perspective, or narrative control remained safeguarded. Collectively, these narratives are viewed as proof of selective adaptation: creators could embrace modifications that enhance cooperation or international comprehensibility while striving to maintain control over decisions they consider culturally significant.

### 3.4 Comparative cases: *TWIN* and *the Medium*

The comparison between *TWIN* and *The Medium* illustrates how contrasting production-governance arrangements generate different configurations of market access, rights allocation, and representational authority, as summarized in Table 3.

**Table 3:** Comparative analysis of *TWIN* and *The Medium.*

Dimension	*TWIN*	*The Medium*
Production configuration	Thailand–Singapore commercial television co-production	Thailand–South Korea film co-production
Access/financing structure	Partner co-financing with Singapore institutional support and territory-based cost sharing	Regional co-production involving GDH and a South Korean partner
Cultural strategy	Universal themes combined with explicitly hyperlocal Thai storytelling	Thai-led culturally specific genre production
Adaptation process	Selective script, protagonist, casting, and cross-market adaptation	Thai-led script development with feedback from the Korean partner
Rights strategy	Thailand rights retained by Thai partner; Singapore/Chinese-speaking rights allocated to Singapore partners; rest-of-world revenue shared	Strong emphasis on Thai IP ownership, domestic distribution, and regional sales interests
Creative authority	Thai showrunner retained Final Say over script, casting, and major production decisions	Thai team led scriptwriting and GDH managed production
Access–Representation configuration	Shared transnational access with explicitly protected local creative authority	Regional collaboration combined with strong Thai production and rights control

#### 3.4.1 *The Medium*: regional collaboration and Thai production authority

*The Medium* demonstrates a framework where international cooperation did not diminish the vital function of the Thai production team. The GDH interview recognized the film as a collaborative production between Thailand and South Korea, which commenced following an initiative from the Korean side to contact GDH. The GDH interview indicates that the Thai crew spearheaded the script development, integrated insights from the Korean collaborator, and then took on significant production duties. This participant’s report indicates that the collaboration enhanced regional production and distribution prospects while enabling the Thai side to maintain significant control over script creation and production. The situation exemplifies a scenario where international cooperation existed alongside significant local manufacturing and rights concerns.

This configuration signifies a crucial aspect of negotiated agency. The collaboration facilitated access to global partnerships and broadened distribution, but the Thai producer retained significant control over the film’s conception and production. The expansive GDH methodology for co-production bolsters this understanding: intellectual property ownership, Thai distribution privileges, and regional sales competencies were regarded as strategic resources instead of rights that are inherently relinquished for international collaboration.

The case therefore illustrates how an intra-Asian manufacturing partnership can merge foreign cooperation with strong local production control. Collaboration expanded access, although representative authority remained significantly entrenched in the local production framework.

#### 3.4.2 *TWIN*: selective adaptation under explicit creative governance

According to the interviewed showrunner, the Thailand–Singapore partnership combined economic and territorial cooperation with institutional support and negotiated market-rights allocation. The showrunner reported establishing conditions concerning creative control before production and retaining authority over major screenplay, casting, and production decisions. This account provides project-specific evidence of how creative authority was negotiated within the *TWIN* production, rather than evidence of a universal co-production model.

The project also involved selective adaptation. The showrunner described changes to the protagonist, internationally coordinated casting, and an effort to develop themes that could travel across national markets while maintaining Thai narrative and cultural specificity. These modifications therefore occurred within a production arrangement in which the Thai creative team retained substantial authority over major creative decisions. The case illustrates how selective adaptation and locally retained creative authority can coexist rather than operating as opposing conditions.

Territorial arrangements followed a similarly differentiated pattern. According to the interview account, the Thai partner retained rights in Thailand, the Singaporean partners were allocated Singapore and Chinese-speaking markets, and revenues from other territories were shared after distribution costs. This territorial structure illustrates how transnational market access can be combined with the retention of strategically important territorial rights. Taken together, the *TWIN* evidence illustrates, rather than establishes as a rule, how transnational financing, territorial allocation, selective adaptation, and protected domains of local creative authority can coexist within a production-governance arrangement.

Collectively, the two cases illustrate contrasting configurations of the Access–Representation Nexus. They suggest that economic interdependence does not necessarily correspond to an equivalent loss of creative or representational authority; however, this interpretation is limited to the cases and interview evidence examined here.

## 4 Discussion

This study examined how screen producers negotiate market access, creative authority, and cultural voice within transnational production governance in a platformized media environment. The findings do not establish platform as an independent causal mechanism. Rather, they show that the observed negotiations are embedded in production-governance arrangements involving financing, territorial rights, intellectual-property allocation, distribution, institutional support, and creative decision-making. The findings therefore qualify, rather than reject, scholarship that identifies digital platforms as powerful infrastructural intermediaries shaping distribution, audience access, financing, and cultural circulation (Couldry and Mejias, 2020; Evens and Donders, 2018; Li, 2022; Napoli, 2019; van Dijck et al., 2018).

This distinction modifies how platformization should be interpreted in relation to producer agencies. The contractual and institutional mechanisms observed in this study—including territorial licensing, rights allocation, co-financing, exclusivity, and negotiated creative authority—are not analytically specific to digital platforms; related arrangements also operate through broadcasters, studios, financiers, co-production systems, and other institutional frameworks (Evens and Donders, 2018; Nieborg and Poell, 2018; Idiz and Poell, 2025; Stelmach, 2026). Platformization is therefore understood here as a context that can intensify and reconfigure existing production-governance relationships, for example by expanding acquisition and distribution pathways, altering the strategic value of territorial and worldwide rights, and increasing the importance of transnational circulation. This interpretation avoids attributing contractual or creative outcomes directly to platforms when the empirical evidence does not establish such causality.

The contribution of the Access–Representation Nexus is consequently not to redefine all production governance as platform governance. Instead, it identifies a recurrent relationship between two domains of negotiation: access architecture, through which producers obtain finance, rights, institutional support, and pathways to circulation; and representation control, through which producers retain, share, or concede authority over culturally consequential creative decisions. These domains are analytically distinct but interdependent. Access to transnational resources may require concessions to contractual or creative areas, while cultural knowledge, professional legitimacy, regional networks, and symbolic capital may strengthen producers’ bargaining positions within specific negotiations (Bourdieu, 1993, 2005). Nexus therefore locates platform actors alongside producers, broadcasters, financiers, distributors, and public agencies within a broader production field rather than treating platforms as the sole source of governance.

### 4.1 Production-governance architecture and bounded authority

The findings indicate that authority within transnational screen production is distributed across multiple institutional and contractual domains rather than concentrated in a single actor. Territorial licensing, co-financing, domestic distribution rights, intellectual-property arrangements, exclusivity, and pre-negotiated creative authority provide different mechanisms through which producers and their partners allocate economic and creative control (Evens and Donders, 2018; Idiz and Poell, 2025; Nieborg and Poell, 2018; Stelmach, 2026). Importantly, these mechanisms are not analytically specific to digital platforms; related arrangements also operate within broadcaster, studio, co-production, financing, and public-institutional frameworks. The relevant issue is therefore not whether contractual power is simply unilateral, but how different forms of authority are distributed and negotiated within production arrangements.

Platformization nevertheless forms part of the changing environment in which these arrangements operate. Digital platforms have become important intermediaries in distribution, acquisition, audience access, and cultural circulation and are associated with an expanded range and scale of transnational pathways available to producers (Couldry and Mejias, 2020; Evens and Donders, 2018; Napoli, 2019; van Dijck et al., 2018). Streaming services may enter production and circulation at different stages—as commissioners, distributors, acquisition partners, or subsequent-window buyers, while expanded cross-border circulation can increase the strategic importance of territorial rights, worldwide rights, licensing windows, and the timing of rights transfer. In various reports from producers and practitioners within the examined domains, streaming services emerged not only as first commissioners but also as acquisition or subsequent distribution collaborators. This cross-interview methodology aligned with contextual industry data about the procurement and dissemination of finalized regional titles (refer to Supplementary Table S1). The discovery is consequently restricted to the production environments analyzed and ought not to be regarded as a universal depiction of all SVoD commissioning frameworks. Platformization is thus regarded not as the source of the governance mechanisms identified, but rather as a wider framework that could amplify or reshape their functioning.

Within the interview corpus and comparative cases, producers employed several strategies to retain strategically important forms of authority. These included territorial licensing, regional co-financing, protection of domestic distribution rights, differentiated revenue and rights arrangements, and the early specification of creative decision-making authority. Such practices reveal an important distinction between economic or intellectual property ownership and creative authority. Some producers accepted shared investment, divided territorial rights, or externally financed production while seeking to protect final decision-making, narrative authority, domestic distribution, or other culturally consequential domains. Financial participation and ownership therefore did not automatically correspond to equivalent control over representation.

The findings consequently suggest that authority in transnational production is multidimensional rather than absolute. A producer may concede control in one domain while retaining substantial authority in another. In one configuration, external financing and market access coexisted with locally retained authority over screenplay development, casting, and production decisions; in another, international collaboration expanded financing and circulation while the local production team remained central to script development and production governance. The analytical significance of these configurations lies not in whether international partners or platforms were present, but in how rights, resources, territorial access, and creative decision-making were allocated among the participating actors.

This distinction clarifies the concept of bounded authority. Producers operating within structurally unequal production environments do not necessarily possess complete economic independence or unrestricted creative autonomy. However, they are neither uniformly subordinated to financiers, distributors, nor platforms. They may instead construct limited but strategically significant domains of authority by defining non-negotiable rights early, segmenting territories, retaining creative decisions, and selectively exchanging other rights for financing or circulation. The Access–Representation Nexus captures this differentiated configuration by showing that access to resources and markets and authority over cultural representation are related but not identical dimensions of production governance. Their relationship must therefore be examined empirically at the level of specific rights, institutional arrangements, and creative decisions rather than inferred from the presence of platform participation alone.

### 4.2 Cultural proximity as negotiable capital

The findings extend existing interpretations of cultural proximity. Prior research has emphasized that audiences may be more receptive to media that resonates with familiar languages, genres, histories, values, and cultural experiences (Chua, 2008; Straubhaar et al., 2021, 2025; Wang, 2025). Research on Asian media circulation has likewise highlighted the importance of regional networks in facilitating transnational cultural exchange (Jin et al., 2023; Kim, 2022).

The present study suggests that cultural proximity can also operate upstream, before audience reception, as a resource within production and negotiation. Through several interviews, participants described regional genre knowledge, culturally grounded narrative expertise, professional relationships, and familiarity with local production contexts as resources that could support project development, partnership formation, and market positioning. These accounts indicate that cultural familiarity may function not only as an audience-side phenomenon but also as a form of production capital.

Bourdieu’s concept of capital conversion helps interpret this pattern (Bourdieu, 1993, 2005). Cultural knowledge, genre expertise, professional credibility, regional networks, and symbolic legitimacy can operate as forms of cultural and symbolic capital. These resources do not eliminate inequalities in finance, infrastructure, or distribution, but the convergence of participant accounts suggests that they may strengthen a producer’s position in particular negotiations. Knowledge of culturally specific genres or local production practices, for example, may provide forms of expertise that external partners cannot easily reproduce.

The term manufactured cultural proximity is used here to describe the deliberate cultivation and mobilization of pre-existing cultural and relational affinities rather than the artificial creation of cultural similarity. Producers may identify, develop, and communicate regional connections through sustained professional relationships, genre knowledge, institutional networks, or culturally grounded storytelling. In this sense, proximity becomes strategically relevant when such resources are translated into partnership credibility, project legitimacy, or negotiating leverage.

The findings also suggest that transnational legitimacy need not depend exclusively on Western recognition. Participants described regional festivals, co-production networks, professional relationships, and intra-Asian market performance as sources of visibility and credibility. These forms of recognition may contribute to symbolic capital in subsequent negotiations, although the present study does not independently establish their effects on audience interpretation or commercial success.

The evidentiary status of this concept should be stated precisely. What the present data demonstrate is that participants possess these resources, invest in them deliberately, and perceive them as consequential in partnership formation — a convergent pattern across informants in three countries. What the data cannot demonstrate, in the absence of contractual records or partner-side accounts, is that these resources caused specific bargaining outcomes. Manufactured cultural proximity is therefore advanced as a mechanism proposition grounded in convergent participant testimony, not as a demonstrated bargaining effect. The closest available evidence of its causal relevance is negative: in the account of one practitioner, the lapse of sustained reciprocal engagement was followed by the closure of a previously productive project pipeline, indicating that the resource is at least perishable and consequential in its absence. Establishing its positive effects on negotiated terms would require access to contracts, deal memoranda, or matched partner-side interviews, which we identify as a priority for future research.

### 4.3 Hyperlocalism, cultural visibility, and the risk of platform capture

Hyperlocalism emerged in interviews as a productive but potentially ambivalent strategy. Several participants described culturally specific narratives, genre traditions, and local knowledge as resources that could differentiate a project within transnational production and circulation. At the same time, the wider literature raises the possibility that market demand for cultural distinctiveness may influence which forms of locality become most legible, promotable, or commercially attractive.

This tension can be interpreted through the concept of platform capture, understood here as a theoretical risk rather than as an independently demonstrated empirical effect. Scholarship on platformization has shown that cultural production may become increasingly shaped by market logics, platform infrastructures, and standardized categories of visibility and circulation (Arsel, 2026; Kim, 2026; Poell et al., 2017). Under such conditions, culturally distinctive material may gain expanded opportunities for transnational circulation while also becoming subject to commercial selection and formatting pressures (Curtin and Sanson, 2017; Marčeta, 2021; Li, 2022; Soeiro et al., 2025).

The interview evidence nevertheless indicates that hyperlocalism should not be understood solely as an externally imposed market strategy. Participants described the active use of cultural specificity as part of their own creative and negotiating practices. In *TWIN*, for example, the interviewed showrunner articulated a “hyperlocal to global” logic in which Thai cultural identity remained important to the project’s transnational positioning. Philippine participants similarly emphasized the importance of retaining culturally grounded narratives rather than continually modifying them in response to financing opportunities.

The analytical issue, therefore, is not whether platforms directly determine the content or meaning of hyperlocalism, but how culturally specific material is negotiated within wider production and distribution environments. Where producers retain substantial authority over narrative voice, cultural meaning, and creative decision-making, local specificity may function as a resource for differentiation and cultural expression. Where such authority is more constrained, the literature suggests a risk that cultural particularity may become simplified or standardized for circulation.

Because this study does not include platform executives, proprietary recommendation data, or direct audience-reception evidence, it cannot determine whether algorithms or platform procurement systems independently produce these outcomes. The contribution of the present findings is more limited: they show how producers perceive and negotiate the value of cultural specificity within production settings shaped by multiple actors, including platforms, financiers, distributors, public institutions, and co-production partners. Throughout this section, visibility dynamics, commercial selection pressures, and platform capture are advanced as theoretically grounded possibilities consistent with the platformization literature and with participants’ stated concerns; none is presented as an effect demonstrated by the present empirical design, which included neither audiences nor platform decision-makers.

### 4.4 Selective adaptation and negotiated cultural voice

The findings complicate the assumption that adaptation for transnational markets necessarily entails cultural capitulation. Previous research has identified the influence of pacing, episode length, genre conventions, character development, formatting, and narrative structure within platformized cultural production (Nieborg and Poell, 2018; Poell et al., 2021). Scholars have also raised concerns that culturally complex material may be simplified to enhance transnational legibility (Kim, 2022; Mirrlees, 2024).

The present findings indicate that adaptation was selective rather than uniform. Participants distinguished between elements they considered open to modification and those they regarded as culturally or creatively consequential. Reported areas of flexibility included casting, protagonist characteristics, production style, format, market positioning, and other elements intended to facilitate collaboration or circulation. By contrast, participants frequently placed greater importance on narrative voice, culturally significant meanings, final decision-making authority, local belief systems, and other elements regarded as central to the project’s cultural or creative integrity.

The contrast between *TWIN* and *The Medium* illustrates this distinction. According to the interviewed showrunner, *TWIN* involved modifications to the protagonist, casting, and other formal elements while retaining substantial Thai authority over major creative decisions. *The Medium*, by contrast, provides an intra-Asian co-production configuration in which collaboration with a South Korean partner coexisted with substantial Thai responsibility for script development and production. These cases illustrate different ways in which adaptation can occur without necessarily eliminating locally retained creative authority.

Creative autonomy should therefore not be evaluated solely according to whether modification occurs. Of greater analytical importance is who authorizes the modification, which elements are altered, why those changes are made, and which domains of cultural or creative authority remain protected. Cultural voice is thus negotiated and differentiated rather than absolute. Producers operate within commercial and institutional constraints, but they may still exercise agency by establishing boundaries around creative and representational domains.

This interpretation also extends the use of Bhabha’s concept of the Third Space. In the present study, negotiation occurs not only at the level of hybrid cultural texts but also through production practices, financing arrangements, territorial-rights allocation, casting, and creative decision-making. The Third Space may therefore be understood as both a cultural and institutional site in which the terms of transnational production are negotiated.

### 4.5 Theoretical contribution of the access–representation nexus

The principal theoretical contribution of this study is the Access–Representation Nexus, which connects the conditions of market access with the allocation of representational authority within transnational production governance. Existing scholarship has often examined platformization, political-economic asymmetry, cultural proximity, creative labour, and cultural representation through partly separate analytical lenses. The Nexus brings these concerns into relation by focusing on how access to resources and circulation interacts with authority over culturally consequential creative decisions.

Access Architecture refers to the financial, legal, institutional, territorial, and distributive arrangements through which producers obtain resources and pathways to markets. Representation Control refers to the differentiated forms of authority through which producers retain, share, or concede control over narrative voice, culturally significant meanings, final decision-making, casting, adaptation, localization, and related creative domains. The two dimensions are analytically distinct but interdependent. Conditions of financing, rights ownership, territorial allocation, and distribution may affect producers’ bargaining positions, while cultural knowledge, professional legitimacy, regional networks, and symbolic capital may strengthen their negotiating capacity in particular production arrangements.

What distinguishes the Access–Representation Nexus from adjacent frameworks is not the topics it addresses but its unit of analysis and the specific relationship it names. Adjacent frameworks each theorize one side of the phenomenon: field theory explains the unequal resources actors bring to negotiation; the Third Space describes the hybrid cultural space in which negotiation occurs; transnational production studies classify collaborations by motivation and institutional form; hybridity and glocalization operate at the level of textual strategy; media political economy specifies structural asymmetry between centres and peripheries; and platformization scholarship theorizes the governing power of a particular category of actor. None of these frameworks, however, specifies how market access and representational authority are settled together — within the same governance arrangement, in the same negotiation, such that movement in one domain is purchased with movement in the other. That co-settlement, and the observable gradient of negotiated positions it produces, is the object the Nexus names. Table 4 summarizes the differentiation.

**Table 4:** Differentiating the Access-Representation Nexus from adjacent frameworks.

Framework	Primary analytical object/unit	What it does not specify	What the Nexus adds
Bourdieu’s field/capital theory	Unequal positions and convertible capitals within a field	How capitals are traded against representational authority inside a specific governance arrangement	The deal-level exchange rate: which capitals convert into which authority instruments, settled before finance
Bhabha’s Third Space	Hybrid cultural space of negotiation and enunciation	The contractual structuring that pre-allocates who may define the terms of hybridity	Shows the “space” is entered with rights already distributed; authority instruments set the boundaries of mixing
Transnational production studies	Motivations and institutional forms of collaboration (project level)	How authority is distributed within collaborations sharing the same motivation	Shifts the unit from project motivation to producer governance architecture
Cultural hybridity/glocalization	Textual strategies mixing global and local elements	The production-governance conditions under which mixing is authorized or refused	Locates hybridity decisions inside authority settlements (final say, adaptation rights)
Media political economy	Structural asymmetry between centres and peripheries	Variation in outcomes among actors occupying the same structural position	Explains within-position variation through deal-level instrument configurations
Platformization scholarship	Infrastructural and market power of platforms as governing actors	Governance mechanisms that predate platforms and operate in broadcaster and treaty systems	Treats platforms as one actor category; platformization intensifies but does not originate the mechanisms

Stated as propositions, the Nexus makes three claims that adjacent frameworks do not make and that future research can test. First, access conditions and representational authority are co-negotiated within a shared production-governance arrangement rather than settled in separate arenas. Second, this co-negotiation produces a gradient of positions — from facilitation and service arrangements, in which representational authority is largely conceded, to full ownership configurations, in which it is retained with self-carried risk — observable across cases and sectors. Third, the instruments that protect representational authority (ownership, pre-agreed final cut, unified final say, funding-source selection) can vary independently of economic or IP ownership, so that creative authority does not simply track capital contribution. Evidence of collaborations in which access and representational settlements were negotiated in isolation from each other, or in which authority tracked capital contribution uniformly across configurations, would count against the framework. It is this specified, testable relationship — rather than a synthesis of the constituent literatures — that constitutes the theoretical contribution.

This relationship develops Bourdieu’s field theory by showing how different forms of capital may be mobilized within production negotiation (Bourdieu, 1993, 2005). Economic asymmetry remains important, but the sampled producers were not positioned solely by financial resources. Cultural expertise, professional reputation, institutional relationships, and control over distinctive narrative resources could also shape negotiating positions within projects.

The framework also extends Bhabha’s concept of the Third Space by locating negotiation within the institutional organization of cultural production. Hybridity and negotiation are visible not only in screen texts but also in financing arrangements, territorial rights, co-production structures, adaptation decisions, and the allocation of creative authority. The relevant “space” of negotiation is therefore simultaneously cultural, contractual, and institutional.

Agency within the production settings examined is consequently best understood as bounded and differentiated rather than absolute. Producers cannot eliminate structural asymmetries in finance, infrastructure, distribution, or institutional power. At the same time, the findings do not support treating them as uniformly subordinate to external partners or platforms. Participants described strategies such as protecting territorial rights, developing regional relationships, mobilizing cultural knowledge, selecting financing sources, and retaining authority over creative domains. These practices can generate limited but strategically important forms of economic and representational authority.

The Access–Representation Nexus therefore does not conceptualize production governance as a simple contest between global platforms and local producers. Platforms constitute one category of actor within a broader production field that also includes producers, broadcasters, financiers, distributors, public agencies, and co-production partners. The framework focuses on how access to finance, institutional support, rights, distribution, and circulation is negotiated alongside authority over cultural representation. Platformization may intensify or reconfigure these relationships, but the underlying governance mechanisms are not specific to digital platforms. The theoretical contribution of the Nexus lies in making visible how access conditions and representational authority are co-negotiated within production arrangements.

### 4.6 Limitations and directions for future research

This study has several limitations that delimit the scope of its findings. First, the interview sample comprised ten information-rich industry informants from Thailand, the Philippines, and Australia. The sample was designed to provide analytical depth and cross-role comparison rather than statistical or regional representativeness. The inclusion of producers, studio practitioners, production and legal specialists, technical professionals, and policy-related actors enabled the study to examine production negotiation from multiple positions within the screen-production field. Nevertheless, this focused sample cannot represent the diversity of production systems across the wider Asia-Pacific region. Major production contexts such as South Korea, Japan, China, India, and Indonesia were not directly represented in the interview sample. The findings should therefore be understood as analytically grounded patterns derived from the institutional contexts examined rather than as generalizations about Asia-Pacific screen production.

Second, the study relies substantially on retrospective professional accounts. Commercial confidentiality and non-disclosure obligations restricted direct access to production contracts, detailed budgets, royalty arrangements, proprietary platform analytics, recommendation systems, and internal platform decision-making. The evidence therefore reveals how producers and other industry actors experienced, described, and interpreted negotiations concerning financing, rights, distribution, and creative authority, but it cannot independently verify every contractual or platform-internal mechanism discussed by participants. Cross-role comparison, comparative case analysis, and documentary or contextual triangulation strengthen the credibility of these accounts, but retrospective testimony remains subject to selective recall and differences in professional interpretation. Accordingly, the study does not infer platform-specific causal effects where direct evidence of platform intervention is unavailable. This restriction applies with particular force to manufactured cultural proximity: the study documents the possession and cultivation of cultural and relational resources, but their causal contribution to specific bargaining outcomes remains a proposition to be tested against contractual and partner-side evidence.

Third, the comparative analysis was limited to *TWIN* and *The Medium*. These productions were selected as analytically illustrative cases because they provide contrasting configurations of transnational financing, territorial rights, institutional support, creative decision-making, cultural adaptation, and distribution. They do not, however, capture the full range of production-governance arrangements across genres, industrial systems, or distribution models. Their culturally specific production contexts may also make questions of cultural proximity, hyperlocalism, and narrative negotiation particularly visible. The cases should therefore be understood as comparative configurations through which the Access–Representation Nexus is examined rather than as representative models of transnational screen production more generally.

Fourth, the study concentrates primarily on producers and other industry decision-makers. It does not directly examine the perspectives of platform executives, all financing and distribution partners, broader production personnel, or audiences. The analysis can therefore explain how participants negotiate the conditions under which cultural content is financed, produced, adapted, and circulated, but it cannot establish how audiences subsequently interpret that content or whether participants’ expectations concerning cultural visibility, recognition, or audience response are realized in practice. Claims concerning audience effects and cultural recognition are therefore treated as participant perceptions or theoretical implications rather than directly observed outcomes.

Future research could extend the Access–Representation Nexus geographically and institutionally. Comparative studies involving additional production centers—including South Korea, Japan, China, India, Indonesia, and other Southeast Asian industries—could examine whether the relationship between Access Architecture and Representation Control differs across dominant, mid-sized, and smaller production systems. Comparative work could also investigate different institutional configurations, including global SVoD services, regional streaming services, broadcasters, public funding bodies, theatrical distribution networks, studios, and independent co-production arrangements. Such research would help identify which features of Nexus travel across production systems and which remain dependent on institutional, legal, or market conditions.

Future studies could also strengthen the evidentiary basis by incorporating anonymized contractual material, territorial-rights documentation, financing records, distribution agreements, and longitudinal observation across project development, production, acquisition, and circulation. Interviews with platform executives, commissioners, distributors, financiers, creative personnel, and other institutional actors would allow production-governance claims to be examined from multiple sides of the negotiation. Audience-reception research could subsequently investigate whether the forms of cultural voice that producers seek to preserve are recognized, interpreted, or contested after circulation. Together, these extensions would move beyond the present study’s emphasis on producer-side negotiation toward a fuller account of how access conditions and representational authority are configured across production and circulation within a platformized media environment.

## 5 Conclusion

This study examined how screen producers negotiate market access, creative authority, and cultural voice within transnational production governance in a platformized media environment. Across the interviews and comparative cases, the findings identified a recurrent “negotiate–adapt” dynamic involving the negotiation of rights and territorial arrangements, the mobilization of culturally grounded knowledge and regional relationships, and selective adaptation of narrative or production elements. Rather than indicating either complete producer autonomy or straightforward external control, these practices reveal differentiated negotiations in which access to financing, institutional support, distribution, and transnational circulation may coexist with efforts to retain authority over culturally consequential creative decisions.

The comparison of *TWIN* and *The Medium* illustrates two different configurations of this relationship. In both cases, transnational collaboration involved the allocation of financing, territorial rights, production responsibilities, and creative authority, but these elements were not distributed in identical ways. The cases therefore suggest that economic interdependence does not necessarily correspond to an equivalent loss of creative or representational authority. Of greater analytical importance is how specific rights and decision-making domains are allocated, which forms of adaptation are accepted, and which aspects of cultural or creative authority producers seek to retain. These interpretations are limited to the cases and production settings examined rather than being general claims about Asia-Pacific screen production.

The Access–Representation Nexus constitutes the study’s principal theoretical contribution. Rather than treating production governance as an extension of platform power, the framework identifies the relationship between the conditions through which producers obtain financial, legal, institutional, territorial, and distributive access and the forms of representational authority they retain, share, or concede. In this respect, the framework extends scholarship on platformization and media political economy, which has demonstrated the infrastructural and intermediary power of digital platforms in shaping access, distribution, and cultural circulation (Couldry and Mejias, 2020; Evens and Donders, 2018; van Dijck et al., 2018). The findings nevertheless indicate that territorial licensing, intellectual-property allocation, financing arrangements, distribution rights, and negotiated creative authority are not analytically specific to digital platforms; related governance mechanisms also operate through broadcasters, studios, financiers, co-production systems, and public-institutional arrangements (Evens and Donders, 2018; Nieborg and Poell, 2018; Idiz and Poell, 2025; Stelmach, 2026).

Platformization is therefore understood in this study as a structural environment that may intensify or reconfigure existing production-governance relationships rather than serving as their sole origin. Digital platforms provide additional pathways for commissioning, acquisition, distribution, and transnational circulation; within this changing environment, the strategic significance of territorial rights, worldwide rights, licensing arrangements, and market access may also shift. However, the evidence does not support platform-specific causal claims concerning proprietary algorithms, internal audience analytics, confidential platform decision-making, or other unobserved mechanisms. The Access–Representation Nexus consequently locates platforms alongside producers, broadcasters, financiers, distributors, public agencies, and co-production partners within a broader field of unequal production relationships.

Cultural agency under these conditions is relational, differentiated, and structurally constrained. Producers cannot eliminate asymmetries in capital, infrastructure, institutional resources, or distribution, but neither are they simply passive recipients of external requirements. Their negotiating capacities depend partly on the resources they can mobilize, including economic resources, professional legitimacy, cultural knowledge, regional networks, and symbolic capital (Bourdieu, 1993, 2005). By negotiating territorial rights, financing arrangements, distribution pathways, culturally consequential meanings, and specific domains of creative authority, producers may construct bounded forms of economic and representational authority within transnational production systems. The governance of global screen culture therefore concerns not only access to resources and circulation but also how representational authority is allocated, which aspects of cultural expression remain negotiable or protected, and under what institutional conditions cultural voices enter wider circuits of communication.

## Data Availability

The raw data supporting the conclusions of this article will be made available by the authors, without undue reservation.
