# Supplementary material for: Negotiating cultural voice in the platform era: the access–representation nexus in Asia-Pacific screen production
Source: Front Sociol. 2026 Oct 14;11:1987870. doi: 10.3389/fsoc.2026.1987870 (PMC13713306; doi:10.3389/fsoc.2026.1987870)
Supplement: Supplementary file 1 [file Table_1.docx]

**Supplementary Table S1. Evidence-Triangulation Matrix**

Central claims are mapped to supporting informants, documentary/contextual corroboration, and evidentiary register. DS = data-supported; PP = participant-perceived/reported; AI = author-inferred. Documentary/contextual material is not treated as independent verification of confidential contractual terms.

| **#** | **Central claim** | **Informant sources / position** | **Documentary or contextual corroboration** | **Register** |
| --- | --- | --- | --- | --- |
| C1 | Rights and territorial arrangements are commonly settled before or alongside financial commitment. | KI-01 (studio executive); KI-03 (art-house producer); KI-04 (showrunner); P01 (independent producer); P02 (regional CEO); A01 (practitioner); A02 (regulatory authority). | Screen Australia International Co-Production Program guidelines; IMDA co-production fund conditions. These materials contextualize the relationship between financing, rights and approval structures. | DS |
| C2 | Official/treaty status can structure market access by conferring national-film benefits and linking finance to production participation. | A02 (regulatory authority); KI-08 (promotion agency); P03 (national film agency). | Screen Australia co-production programme materials and statistics; Telefilm Canada Philippines treaty materials; Australian Office for the Arts treaty materials. | DS |
| C3 | Territorial licensing and rights allocation are used to manage IP value, distribution authority and transnational buyer relationships. | KI-01 (studio); KI-04 (showrunner); P01 (independent producer); A01 (practitioner). | Public co-production and release records provide contextual corroboration. The specific TWIN territorial allocation is participant-reported; no public contract was available. KOFIC records corroborate The Medium's Korean release context but not confidential rights terms. | DS / PP (project-specific instrument details) |
| C4 | Manufactured cultural proximity refers to the deliberate cultivation and mobilization of pre-existing cultural and relational affinities that may function as a production resource and strengthen negotiations. | A01 (long-term guanxi/sister-state investment); KI-02 (regional genre affinity); P02 (regional network building); KI-08 (sustained strategic relationship building). | Sister-state programme records and regional market evidence provide contextual corroboration for sustained cross-border ties and demand; they do not independently establish bargaining effects. | DS / AI |
| C5 | Creative authority is multidimensional and can be protected through different instruments that do not necessarily track economic or IP ownership. | KI-01 (ownership); KI-02 (pre-agreed final cut); KI-03 (funding-source curation); KI-04 (final say); P02 (narrative authority). | No public contracts were available. The broader multidimensional-authority claim is supported by convergent testimony across practitioners; precise instrument details remain participant-reported. | DS / PP (instrument details) |
| C6 | Participants described hyperlocal distinctiveness as a potential source of cultural differentiation and negotiating value, but the study does not establish a causal effect on bargaining power or market performance. | KI-01 (craft-first philosophy); KI-02 (belief-system/genre signature); KI-04 (partners request local distinctiveness); P02 (cultural-narrative principle); KI-08 (creative-partner agenda). | Regional admissions and market records demonstrate audience demand for selected Thai-origin titles, but these records evidence purchasing behaviour rather than bargaining effects or cultural interpretation. | PP / AI |
| C7 | In the sampled fields, streaming services appeared not only as originating commissioners but also as acquisition or later-window distribution partners. | KI-01 (finished-work sales); KI-03 (late-window acquisition); KI-04 (own-platform and external-platform deals); P01 (worldwide-rights preservation for streamers). | Trade reporting on Southeast Asian streaming acquisition of completed Thai titles; regional streaming-demand reports (e.g., Media Partners Asia; Omdia). | DS |
| C8 | Negative and boundary cases show that relational access can deteriorate and that maintaining representational principles may carry economic costs. | KI-02 (project collapse linked to political climate); A01 (reciprocity lapse and access closure); P02 (funding opportunity lost after refusing narrative tailoring). | Public bilateral-relations chronology is consistent with the reported project-collapse context; other project-level consequences remain participant-reported. | DS / PP |
| C9 | Platformization may intensify or reconfigure, but does not originate, the production-governance mechanisms observed; comparable mechanisms predate streaming and operate in broadcaster, studio, treaty and co-production systems. | A02 (treaty-system mechanisms); KI-08 (emerging streaming obligations/partnerships); KI-04 (broadcaster-anchored governance). | Australian streaming local-content legislation; Screen Australia programme rules and treaty materials predating the streaming era. | DS / AI (theoretical framing) |
| C10 | Market access and representational authority are analytically distinct but relational dimensions that are negotiated within shared production-governance arrangements, producing different negotiated positions (the Access-Representation Nexus). | Interpretive synthesis across all ten informants and the TWIN/The Medium case comparison. | Constituent claims are corroborated as above. The Nexus itself is an author-level theoretical integration rather than a directly observed contractual fact. | AI (built on DS/PP components) |

**Notes.** Informant codes use the analytical source scheme T = Thailand, P = Philippines, and A = Australia. Figure 1 shows the coding hierarchy; Supplementary Table S2 provides the complete 34-code mapping.
