# Supplementary material for: Negotiating cultural voice in the platform era: the access–representation nexus in Asia-Pacific screen production
Source: Front Sociol. 2026 Oct 14;11:1987870. doi: 10.3389/fsoc.2026.1987870 (PMC13713306; doi:10.3389/fsoc.2026.1987870)
Supplement: Supplementary file 2 [file Table_2.docx]

**Supplementary Table S2. Full Coding Hierarchy of the Access-Representation Nexus**

The final coding structure comprises 34 first-order codes organized into nine second-order themes and two aggregate dimensions: Access Architecture (five themes, 20 codes) and Representation Control (four themes, 14 codes). Negotiated agency is treated as the relational process through which participants navigate the two dimensions, not as a third aggregate dimension.

| **Aggregate dimension** | **Second-order theme** | **First-order code** | **Operational meaning** | **Informant coverage** | **Illustrative evidence** |
| --- | --- | --- | --- | --- | --- |
| Access Architecture | A1. Rights and territory settlement | Rights settled before financial commitment | Rights/territorial architecture clarified before final financial commitment. | KI-01, KI-03, KI-04, P01, P02, A01, A02 (n=7) | Studio account: IP ownership and Thai distribution rights treated as non-negotiable. |
| Access Architecture | A1. Rights and territory settlement | Territorial split design | Allocation of territorial distribution or exploitation rights among partners. | KI-01, KI-04, A01, A02 (n=4) | Home market to the Thai side; partner home and Chinese-language markets to the partner; rest-of-world shared after a fixed selling commission. |
| Access Architecture | A1. Rights and territory settlement | Home-market retention | Retention of domestic/home-market rights by the local partner. | KI-01, KI-04 (n=2) | Thai distribution rights retained in every deal. |
| Access Architecture | A1. Rights and territory settlement | Global-rights preservation strategy | Preservation of worldwide-rights value by avoiding premature territorial fragmentation. | P01, KI-01 (n=2) | Avoid pre-selling specific territories so that worldwide streaming value remains intact. |
| Access Architecture | A2. Funding and incentive navigation | Self-financing as leverage | Use of own capital to reduce dependence and strengthen bargaining over rights/control. | KI-01, KI-04 (n=2) | The studio finances most of its slate itself, placing great importance on IP ownership as its most asset. |
| Access Architecture | A2. Funding and incentive navigation | Multi-country grant assembly | Combination of public grants or funds from multiple jurisdictions for one project. | KI-03, P01, P02 (n=3) | European public funds and a Singaporean grant assembled per project. |
| Access Architecture | A2. Funding and incentive navigation | Incentive economics (rebates, offsets, QAPE) | Use and assessment of rebates, offsets, qualifying expenditure and related incentives. | A01, A02, KI-08, P03 (n=4) | Regulatory account of producer-offset certificates and co-production approvals. |
| Access Architecture | A2. Funding and incentive navigation | State co-production fund conditions | Funding conditions, milestones and approval requirements attached to public co-production support. | KI-03, KI-04, P01, P03, A02 (n=5) | Agency account of tranche-based public-fund disbursement. |
| Access Architecture | A3. Relational infrastructure | Festival and market networking | Relationship formation and project development through festivals, markets and industry events. | KI-02, KI-03, P01, P02, A02, KI-08 (n=6) | Regulatory account identifying festivals/markets as starting points for co-production relationships. |
| Access Architecture | A3. Relational infrastructure | Sustained partner cultivation | Long-term maintenance of cross-border professional relationships and reciprocity. | KI-01, KI-02, P02, A01 (n=4) | Producer account describing durable partnerships as long-term relationships requiring mutual fit. |
| Access Architecture | A3. Relational infrastructure | Bicultural liaison personnel | Use of bilingual/bicultural intermediaries to bridge production and cultural contexts. | KI-04, A01 (n=2) | A Chinese cultural liaison inside the team described as a condition of success. |
| Access Architecture | A3. Relational infrastructure | Institutional training networks | Professional relationships and capability are built through training programmes and alumni networks. | KI-03, P01, P02 (n=3) | Regional capability is built through international training-institution networks. |
| Access Architecture | A4. Institutional frameworks and gaps | Treaty mechanics and joint approval | Formal treaty/co-production approval mechanisms that confer national status and benefits. | A02, KI-08, P03 (n=3) | Regulatory account: approved co-productions can qualify as national films in participating territories. |
| Access Architecture | A4. Institutional frameworks and gaps | Operating without treaty frameworks | Cross-border production undertaken outside formal treaty structures. | KI-01, KI-02, KI-03, KI-04, A01 (n=5) | All China-facing co-production conducted outside the treaty because the projects did not fit the model. |
| Access Architecture | A4. Institutional frameworks and gaps | Agency incentive programmes | Government or agency programmes offering grants, incentives or staged support. | P03, A02, KI-08 (n=3) | Three tranches based on milestones; roughly twelve grantees per year since 2020. |
| Access Architecture | A4. Institutional frameworks and gaps | Perceived competitive disadvantage | Participant perceptions that domestic incentives or policy settings are less competitive. | P02, P03, A02 (n=3) | Agency account explicitly questioning competitiveness of available incentives. |
| Access Architecture | A5. Distribution pathway design | Theatrical territorial sales | Sale of completed works territory by territory for theatrical release. | KI-01, KI-02, A02 (n=3) | In-house sales teams and global agencies sell finished works territory by territory. |
| Access Architecture | A5. Distribution pathway design | Festival-first windowing | Use of festival premieres as an early circulation window before later sales/acquisitions. | KI-03, P01 (n=2) | Festival premiere as the gateway to territorial sales and late platform windows. |
| Access Architecture | A5. Distribution pathway design | Platforms as late-window buyers | Streaming platforms entering after production as acquisition or later-window buyers. | KI-01, KI-03, KI-04, P01 (n=4) | Platforms encountered as end-of-chain buyers without creative authority over the projects. |
| Access Architecture | A5. Distribution pathway design | Alternative and community distribution | Use of non-mainstream, community or digital pathways to reach audiences. | P02 (n=1) | Producer accounts describing digital/community pathways to bypass theatrical limitations. |
| Representation Control | R1. Creative authority instruments | IP ownership as sovereignty | Retention of IP ownership as a strategic form of continuing economic/creative authority. | KI-01, KI-04, P01, P02 (n=4) | IP is viewed as the most asset because it keeps generating value; ownership is non-negotiable. |
| Representation Control | R1. Creative authority instruments | Pre-agreed final cut | Final-cut authority explicitly agreed before production or financing is completed. | KI-02, KI-03 (n=2) | Director account reporting final creative say agreed from the outset while still considering partner input. |
| Representation Control | R1. Creative authority instruments | Single decision-maker authority | Concentration of ultimate creative decision authority in one designated role. | KI-02, KI-04 (n=2) | Showrunner account reporting “Final Say” over major creative decisions. |
| Representation Control | R1. Creative authority instruments | Funding-source curation | Selection or avoidance of funding sources according to attached creative conditions. | KI-03, P01 (n=2) | The public funding the company works with does not interfere with content or final cut at all. |
| Representation Control | R2. Narrative authority and cultural voice | Narrative authority as principle | Protection of narrative voice as a non-negotiable creative principle. | KI-02, KI-04, P02 (n=3) | Producer account defining narrative voice as a core condition, not only a financing issue. |
| Representation Control | R2. Narrative authority and cultural voice | Hyperlocal distinctiveness as asset | Use of culturally specific/local distinctiveness as a creative and strategic resource. | KI-01, KI-02, KI-04, P02 (n=4) | Partners ask for local distinctiveness, done one’s own way, because that is what differentiates the work. |
| Representation Control | R2. Narrative authority and cultural voice | Genre and belief-system strategy | Mobilization of culturally rooted genre knowledge and belief systems in project positioning. | KI-01, KI-02, P02 (n=3) | Horror grounded in Thai and Southeast Asian belief-worlds as the signature partners come for. |
| Representation Control | R3. Negotiated adaptation | Flexible versus non-negotiable elements | Differentiation between adaptable formal elements and protected cultural/creative elements. | KI-01, KI-02, KI-04, P02 (n=4) | Casting, pacing and format open to input; ownership, voice and final authority protected. |
| Representation Control | R3. Negotiated adaptation | Casting and language calibration | Adjustment of casting and language choices across markets while managing local meaning. | KI-04, A01 (n=2) | Mixed-territory casting; a First Nations lead placed within a foreign-originated drama. |
| Representation Control | R3. Negotiated adaptation | Facilitation mode (ceded representation) | Service/facilitation role in which representational authority remains with an originating partner. | A01, KI-08 (n=2) | Practitioner account emphasizing bespoke service models where originating financiers retain rights. |
| Representation Control | R3. Negotiated adaptation | Labour-standard clauses | Adoption of labour, safety or production standards through contractual/institutional requirements. | KI-03, KI-04 (n=2) | The 12-hour-per-day standard and on-set health and safety care followed as the highest standard among contracting countries. |
| Representation Control | R4. Boundary conditions and negative cases | Political collapse of projects | Project failure or withdrawal associated with changing political/bilateral conditions. | KI-02, A01 (n=2) | A planned China collaboration died with a change in the political climate. |
| Representation Control | R4. Boundary conditions and negative cases | Reciprocity lapse and access closure | Loss of market/project access when reciprocal relationships are not maintained. | A01 (n=1) | When visits and reciprocity lapsed, the pipeline stalled — the relationship is the market. |
| Representation Control | R4. Boundary conditions and negative cases | Costs of representational principles | Economic or access costs incurred when producers refuse to compromise cultural/narrative principles. | P02 (n=1) | Producer account reporting lost opportunities rather than sacrificing the cultural narrative. |

**Notes.** Informant coverage and illustrative evidence are drawn from the project coding matrix and excerpt log. The source coding frame contains 20 Access Architecture codes and 14 Representation Control codes. Where illustrative evidence is summarized, it is a concise paraphrase of the underlying source excerpt rather than a new empirical claim.
