# Supplementary material for: Negotiating cultural voice in the platform era: the access–representation nexus in Asia-Pacific screen production
Source: Front Sociol. 2026 Oct 14;11:1987870. doi: 10.3389/fsoc.2026.1987870 (PMC13713306; doi:10.3389/fsoc.2026.1987870)
Supplement: Supplementary file 3 [file Table_3.docx]

**Supplementary Table S3. Code Accrual by Interview: Empirical Basis for the Analytical-Sufficiency Judgement**

This table documents, for each interview in chronological order of data collection, the first-order codes first documented at that interview, derived from the coding-presence records of the project coding matrix (Supplementary Table S2 provides the full 34-code hierarchy). A code is counted at its first documented occurrence; subsequent interviews in which the same code was also present contributed additional instances, refinements of code boundaries, and institutional detail, but are not counted again. All thirty-four first-order codes in the final coding frame had been established by the eighth interview (P02); the ninth and tenth interviews (P03, A03) introduced no new first-order codes and no new second-order themes. All nine second-order themes were established by the second interview.

| **Interview** | **Case ID** | **Country / context** | **New first-order codes documented at this interview** | **New codes (n)** | **Cumulative codes (of 34)** | **Cumulative themes (of 9)** |
| --- | --- | --- | --- | --- | --- | --- |
| 1 | T01 | Thailand — GDH559 | Rights settled before financial commitment; Territorial split design; Home-market retention; Global-rights preservation strategy; Self-financing as leverage; Sustained partner cultivation; Operating without treaty frameworks; Theatrical territorial sales; Platforms as late-window buyers; IP ownership as sovereignty; Hyperlocal distinctiveness as asset; Genre and belief-system strategy; Flexible versus non-negotiable elements | 13 | 13 | 8 |
| 2 | T02 | Thailand — Director / filmmaker | Festival and market networking; Pre-agreed final cut; Single decision-maker authority; Narrative authority as principle; Political collapse of projects | 5 | 18 | 9 |
| 3 | T03 | Thailand — Independent art-house producer | Multi-country grant assembly; State co-production fund conditions; Institutional training networks; Festival-first windowing; Funding-source curation; Labour-standard clauses | 6 | 24 | 9 |
| 4 | P01 | Philippines — Independent producer | — (no new codes; additional instances and refinements only) | 0 | 24 | 9 |
| 5 | T04 | Thailand — One D Original (showrunner) | Bicultural liaison personnel; Casting and language calibration | 2 | 26 | 9 |
| 6 | A02 | Australia — Screen Australia | Incentive economics (rebates, offsets, QAPE); Treaty mechanics and joint approval; Agency incentive programmes; Perceived competitive disadvantage | 4 | 30 | 9 |
| 7 | A01 | Australia — 57 Films | Facilitation mode (ceded representation); Reciprocity lapse and access closure | 2 | 32 | 9 |
| 8 | P02 | Philippines — Zero House Multimedia | Alternative and community distribution; Costs of representational principles | 2 | 34 | 9 |
| 9 | P03 | Philippines — FDCP (agency) | — (no new codes; additional instances and refinements only) | 0 | 34 | 9 |
| 10 | A03 | Australia — Ausfilm | — (no new codes; additional instances and refinements only) | 0 | 34 | 9 |

*Note. Interview numbering follows the chronological order of data collection recorded in the fieldwork log: T01 (21 July 2025), T02 (6 August 2025), and T03 (10 December 2025), followed by P01 (18 January 2026), T04 (30 January 2026), A02 (Screen Australia, 8 April 2026), A01 (57 Films, 13 April 2026), P02 (14 April 2026), P03 (FDCP, 12 May 2026), and A03 (Ausfilm, 19 May 2026). Coding presence is drawn from the project coding matrix (34 first-order codes × 10 informant cases; 102 code–case presences). The sufficiency judgement reported in Section 2.3 rests on this record and is advanced as the empirical basis for ceasing recruitment, not as a claim of exhaustive thematic saturation.*
